# Analysis of risk factors for urinary tract infection and bleeding after retrograde flexible ureteroscopy for stone removal

**DOI:** 10.3389/fsurg.2025.1573485

**Published:** 2025-04-23

**Authors:** Tao Guo, Jiaen Zhang, Wenzhi Gao, Yixiang Ma

**Affiliations:** ^1^ Department of Emergency, Peking University First Hospital, Beijing, China; ^2^ Department of Urology, Peking University First Hospital, Beijing, China; ^3^ Department of Urology, Peking University First Hospital-Miyun hospital, Beijing, China

**Keywords:** retrograde flexible ureteroscopy, infection, bleeding, stone size, operation time

## Abstract

**Objective:**

This study aimed to explore the risk factors for urinary tract infection (UTI) and bleeding after retrograde flexible ureteroscopy for stone removal, in order to prevent these complications and improve surgical outcomes.

**Methods:**

A retrospective analysis was conducted on 214 patients who underwent retrograde flexible ureteroscopy for kidney stones and ureteral stones from January 2015 to August 2022, with 135 patients having complete data. Clinical data, perioperative data, and stone characteristics were collected. Univariate and multivariate logistic regression analyses were performed to identify risk factors for UTI and bleeding after retrograde flexible ureteroscopy for stone removal.

**Results:**

The UTI rate after retrograde flexible ureteroscopy for stone removal was 8.15% (11/135), and the bleeding rate was 11.85% (16/135). Factors such as length of hospital stay (*p* = 0.034), stone size (*p* < 0.001), and preoperative creatinine (*p* = 0.016) were identified as risk factors for UTI after retrograde flexible ureteroscopy. Stone size (*p* = 0.004) was an independent risk factor for post-operative UTI. Stone size (*p* < 0.001), operation time (*p* < 0.001), and preoperative creatinine (*p* = 0.023) were risk factors for bleeding after retrograde flexible ureteroscopy. Stone size (*p* < 0.001) and operation time (*p* = 0.024) were independent risk factors for post-operative bleeding.

**Conclusion:**

Stone size is an independent risk factor for UTI after retrograde flexible ureteroscopy for stone removal, while both stone size and operation time are independent risk factors for bleeding after the procedure.

## 1 Introduction

The incidence and prevalence of upper urinary tract stones have been increasing globally (1–3). Common treatment methods for upper urinary tract stones include extracorporeal shock wave lithotripsy (ESWL), ureteroscopy, percutaneous nephrolithotomy (PCNL), and, in some cases, open surgery (4–6). With advancements in medical technology for endourological procedures, retrograde flexible ureteroscopy (R-FURS) for stone removal has become widely used. Its high efficacy and low complication rate in treating kidney stones up to 20 mm in diameter make it a preferred option for upper urinary tract stones (7).

The minimally invasive nature, precision, and low complication rate of retrograde ureteroscopy have expanded its indications (8). Although the number of PCNL procedures has remained stable over the past few decades, ureteroscopic procedures have significantly increased compared to ESWL (9). As an endoscopic surgery, retrograde ureteroscopy has fewer complications, but there are still some risks, with the complication rate around 10%–15%, most of which are Clavien grade II or lower (10). Urinary tract infection (UTI) and bleeding, as the most common complications, deserve particular attention (11).

Exploring the risk factors for UTI and bleeding after R-FURS for stone removal is crucial for identifying potential risks and taking effective preventive measures. This will help reduce the incidence of post-operative UTI and bleeding and provide valuable references for clinicians during diagnosis and treatment.

## 2 Materials and methods

### 2.1 Study population

**Figure 1:**
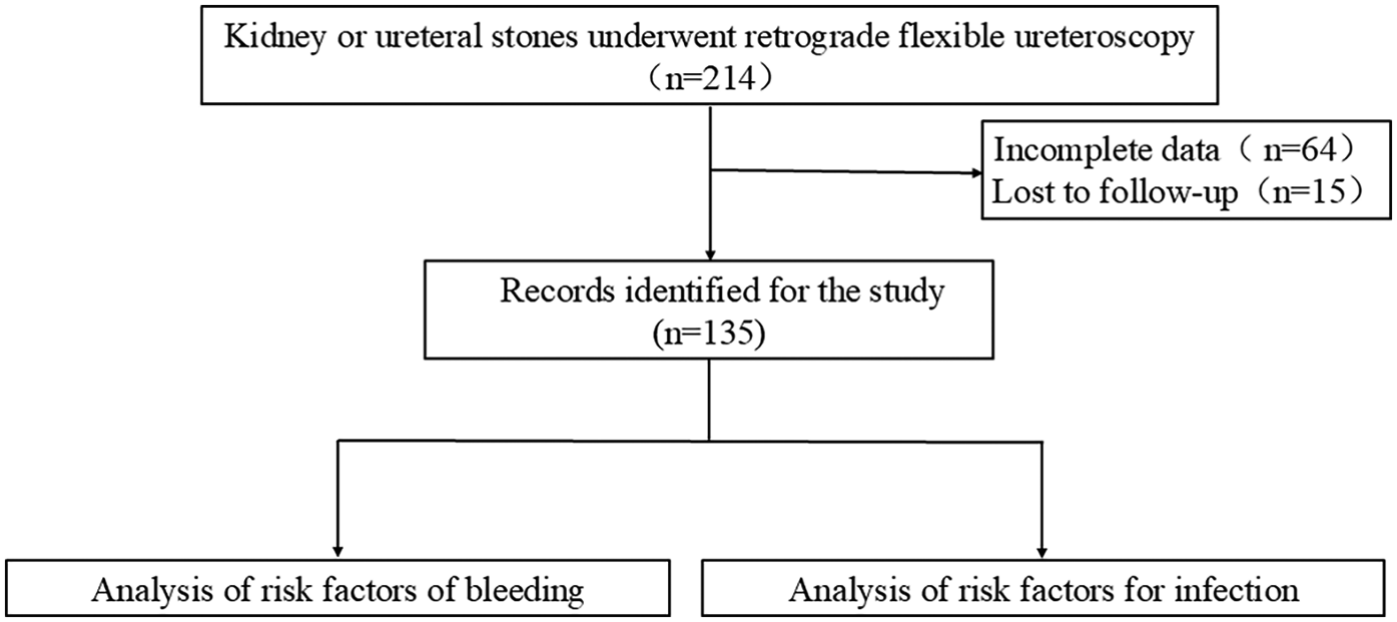
Research steps flowchart for this study.

Between January 2015 and August 2022, 214 patients with kidney and ureteral stones underwent R-FURS at the Department of Urology, Peking University First Hospital-Miyun hospital (Figure 1). Among them, 135 patients had complete data, including 14 patients with ureteral stones and 121 with kidney stones. Clinical data, perioperative data, and stone characteristics were collected.

Preoperative and postoperative day 1 hemoglobin levels were recorded for each patient. Based on the decrease in hemoglobin, patients were divided into two groups: the bleeding group (hemoglobin decrease ≥20 g/L) and the non-bleeding group (hemoglobin decrease <20 g/L). The decrease in hemoglobin (g/L) was calculated as the preoperative hemoglobin level minus the postoperative day 1 hemoglobin level.

Urine culture results within one week post-surgery were also collected. The presence of urinary tract irritation symptoms combined with a positive urine culture (>10^5^ UFC/ml) is defined as a UTI. Patients were divided into the infection group (positive urine culture) and the non-infection group (negative urine culture).

Inclusion Criteria: (a) Age ≥18 years; (b) Preoperative ultrasound, intravenous pyelography, or urinary system CT confirming kidney or ureteral stones; (c) Complete clinical data. Exclusion Criteria: (a) Abnormal coagulation function; (b) Patients with cardiac or pulmonary dysfunction unable to undergo surgery; (c) Patients unable to comply with the study; (d) Patients with abnormal routine urine tests during outpatient examinations.

Clinical variables included gender, age, body mass index (BMI), hypertension, diabetes, coronary artery heart disease (CHD), pulmonary disease, stone location, stone size, operation time, intraoperative bleeding, length of hospital stay, preoperative creatinine, preoperative sodium, preoperative potassium, preoperative calcium, preoperative systolic and diastolic blood pressure. The study aimed to explore the risk factors for UTI and bleeding in patients undergoing R-FURS for kidney and ureteral stones.

This study adhered to the principles of the Declaration of Helsinki (2013 revision) and was approved by the Ethics Committee of Peking University First Hospital-Miyun Hospital. Informed consent was waived for the retrospective analysis.

### 2.2 Surgical technique

After general anesthesia, the patient was placed in the lithotomy position and the area was routinely disinfected and draped. Lidocaine was used to lubricate and anesthetize the urethra. A ureteroscope was inserted through the external urethral orifice to examine the bladder and locate the ureteral orifice. A nickel-titanium guidewire was inserted through the affected ureteral orifice, and the ureteroscope was removed. A F12 ultra-slick catheter was left in place, and the ureteroscope was reintroduced over the guidewire to examine the ureter up to the renal pelvis. After removal of the rigid scope, an F11 ureteral sheath was inserted over the guidewire, followed by the flexible ureteroscope, to examine the renal pelvis and calyces. The stone is fragmented using a holmium laser (with an energy of 0.8–2.0 joules and a frequency of 10–20 hertz), and the fragments are suctioned into the sheath by negative pressure. Larger stones were removed with an N-Gage stone basket, while smaller stone fragments were left for spontaneous passage. The renal pelvis and calyces were re-examined, and if no stones larger than 2 mm were found, the ultra-slick guidewire was left in place, and the ureteroscope and sheath were removed. A F47 double-J stent stent was placed in the ureter, and a three-way catheter was retained for drainage.

### 2.3 Statistical analysis

Data management was performed using Excel (2019 version), and statistical analysis was conducted using SPSS (version 22.0). Quantitative variables such as age, BMI, stone size, operation time, intraoperative bleeding, length of hospital stay, preoperative creatinine, preoperative sodium, potassium, preoperative calcium, and blood pressure (systolic and diastolic) were analyzed. Qualitative variables, including gender, diabetes, hypertension, CHD, pulmonary disease, and stone location, were also analyzed. Normally distributed data were expressed as mean ± standard deviation, and non-normally distributed data were expressed as median (range). *T*-tests were used for normally distributed variables, and Mann–Whitney *U*-tests were used for non-normally distributed variables. Fisher's exact test was used to analyze categorical variables. Univariate binary logistic regression (*p* < 0.05) and multivariate logistic regression (*p* < 0.05) were used to analyze the risk factors for UTI and bleeding.

## 3 Results

### 3.1 Patient baseline data

The patients' ages ranged from 22 to 82 years, with a mean age of 51.99 ± 12.93 years. There were 87(64.44%) male patients and 48(35.56%) female patients. The average stone size was 1.45 ± 0.77 cm, the mean hospital stay was 6.70 ± 2.06 days, the average surgical time was 89.87 ± 43.54 min, and the intraoperative blood loss was 5.18 ± 8.68 ml. The preoperative serum creatinine was 78.64 ± 44.38 mmol/L. The incidence of postoperative UTI was 8.15% (11/135), and the incidence of postoperative bleeding was 11.85% (16/135).

### 3.2 Risk factors for postoperative UTI after R-FURS for stone removal

The clinical data of patients infection group and non-infection group after R-FURS are shown in Table 1. The infection group had an average stone size of 2.57 ± 0.99 cm, while the non-infection group had an average stone size of 1.35 ± 0.66 cm, with a statistically significant difference between the two groups (*p* = 0.011). The infection group had a mean hospital stay of 8.09 ± 3.34 days, while the non-infection group had 6.58 ± 1.85 days, showing a statistically significant difference (*p* = 0.020). The infection group had an intraoperative blood loss of 18.91 ± 14.62 ml, compared to 3.95 ± 9.48 ml in the non-infection group, with a statistically significant difference (*p* = 0.005). The preoperative serum creatinine level in the infection group was 140.00 ± 123.28 mmol/L, while in the non-infection group, it was 73.61 ± 23.47 mmol/L, with a statistically significant difference (*p* < 0.001).

**Table 1:** Comparison between the infection group and the non-infection group after retrograde ureteroscopy with flexible ureteroscope for stone removal.

Variable	Total	Infection group	Non-infection group	*p*-value
Patients, *n* (%)	135	11 (2.15)	124 (91.85)	
Mean age (years)	51.99 ± 12.93	54.82 ± 13.86	51.74 ± 12.82	0.653
BMI (kg/m^2^)	25.86 ± 3.57	24.42 ± 3.97	25.99 ± 3.50	0.257
Gender, *n* (%)				0.549
Male	87 (64.44)	8 (72.73)	79 (63.71)	
Female	48 (35.56)	3 (27.27)	45 (36.29)	
Hypertension, *n* (%)				0.91
Yes	47 (34.81)	4 (36.36)	43 (34.68)	
No	88 (65.19)	7 (63.64)	81 (65.32)	
Diabetes mellitus, *n* (%)				0.723
Yes	31 (22.96)	3 (27.27)	28 (22.58)	
No	104 (77.04)	8 (72.73)	96 (77.42)	
CHD, *n* (%)				0.824
Yes	10 (7.41)	1 (9.09)	9 (7.26)	
No	125 (92.59)	10 (90.91)	115 (92.74)	
Pulmonary disease, *n* (%)				0.765
Yes	1 (0.74)	2 (18.18)	1 (0.81)	
No	134 (99.26)	9 (81.82)	123 (99.19)	
Brain disease, *n* (%)				0.021
Yes	6 (4.44)	2 (18.18)	4 (3.22)	
No	129 (95.56)	9 (81.82)	120 (96.77)	
Stone location, *n* (%)				0.885
Kidney stones	121 (89.63)	10 (90.91)	111 (89.52)	
Ureteral stones	14 (10.37)	1 (9.09)	13 (10.48)	
Stone size (Maximal diameter, cm)	1.45 ± 0.77	2.57 ± 0.99	1.35 ± 0.66	0.011
Length of hospital stay (day)	6.70 ± 2.06	8.09 ± 3.34	6.58 ± 1.85	0.201
Operation time (min)	89.87 ± 43.54	163.73 ± 17.92	59.27 ± 19.59	0.705
Intraoperative blood loss (ml)	5.18 ± 8.68	18.91 ± 14.62	3.95 ± 9.48	0.005
Preoperative serum creatinine (umol/L)	78.64 ± 44.38	140.00 ± 123.28	73.61 ± 23.47	<0.001
Preoperative sodium (mmol/L)	140.24 ± 1.73	139.57 ± 2.03	140.30 ± 1.69	0.194
Preoperative potassium (mmol/L)	4.29 ± 3.18	4.12 ± 0.32	4.31 ± 3.30	0.7
Preoperative calcium (mmol/L)	2.37 ± 0.30	2.30 ± 0.21	2.38 ± 0.31	0.798
Preoperative systolic blood pressure (mmHg)	127.41 ± 8.17	125.36 ± 7.14	127.59 ± 8.23	0.314
Preoperative diastolic blood pressure (mmHg)	77.74 ± 6.05	77.82 ± 5.84	77.73 ± 6.07	0.727

BMI, body mass index; CHD, oronary heart disease.

Univariate logistic regression identified length of hospital stay (*p* = 0.034), stone size (*p* < 0.001), and preoperative serum creatinine (*p* = 0.016) as risk factors for postoperative infection after R-FURS. Multivariate logistic regression analysis revealed that stone size (*p* = 0.004) was an independent risk factor for postoperative infection (Table 2).

**Table 2:** Univariate and multivariate logistic regression analysis for UTI.

Variable	Univariate			Multivariate		
	OR	95% CI	*p*-value	OR	95% CI	*p*-value
Mean age	0.019	0.970–1.070	0.451			
BMI	−0.145	0.711–1.053	0.149			
Hypertension	−0.074	0.258–3.351	0.910			
Diabetes mellitus	−0.251	0.193–3.129	0.723			
CHD	−0.245	0.090–6.818	0.824			
Stone location	−0.083	0.106–7.954	0.885			
Length of hospital stay	0.250	1.019–1.617	0.034	0.098	0.814–1.030	0.527
Stone size	1.327	1.962–7.240	<0.001	1.062	1.397–5.986	0.004
Preoperative serum creatinine	0.021	1.004–1.040	0.016	0.011	0.992–1.030	0.252
Preoperative sodium	−0.246	0.535–1.142	0.203			
Preoperative potassium	−0.028	0.706–1.338	0.862			
Preoperative calcium	−2.412	0.001–10.503	0.321			
Preoperative systolic blood pressure	−0.036	0.889–1.047	0.386			
Preoperative diastolic blood pressure	0.002	0.906–1.109	0.965			

### 3.3 Risk factors for postoperative bleeding after R-FURS for stone removal

The clinical data of patients bleeding group and non-bleeding after R-FURS are shown in Table 3. The bleeding group had an average stone size of 3.02 ± 0.84 cm, while the non-bleeding group had 1.24 ± 0.45 cm, with a statistically significant difference between the two groups (*p* < 0.001). The bleeding group had an average surgical time of 115.19 ± 46.74 min, compared to 61.40 ± 26.82 min in the non-bleeding group, showing a statistically significant difference (*p* < 0.001). The bleeding group had an intraoperative blood loss of 30.00 ± 16.49 ml, while the non-bleeding group had 1.83 ± 1.31 ml, with a statistically significant difference (*p* < 0.001). The preoperative serum creatinine level in the bleeding group was 116.63 ± 105.04 mmol/L, compared to 73.41 ± 22.20 mmol/L in the non-bleeding group, with a statistically significant difference (*p* < 0.001).

**Table 3:** Comparison between the bleeding group and the non-bleeding group after retrograde ureteroscopy for stone removal.

Variable	Bleeding group	Non-bleeding group	*p*-value
Patients, *n* (%)	16 (11.85)	119 (88.15)	
Mean age (years)	55.25 ± 9.38	52.13 ± 13.03	0.882
BMI (kg/m^2^)	25.65 ± 2.07	26.04 ± 3.45	0.100
Gender, *n* (%)			0.446
Male	9 (56.25)	78 (65.55)	
Female	7 (43.75)	41 (34.45)	
Hypertension, *n* (%)			0.750
Yes	5 (31.25)	42 (35.29)	
No	11()68.75	77 (64.71)	
Diabetes mellitus, *n* (%)			0.670
Yes	3 (18.75)	28 (23.53)	
No	13 (81.25)	91 (76.47)	
CHD, *n* (%)			0.851
Yes	1 (6.25)	9 (7.56)	
No	15 (93.75)	110 (92.44)	
Pulmonary disease, *n* (%)			0.713
Yes	0 (0)	1 (0.84)	
No	16 (100)	118 (99.16)	
Stone location, *n* (%)			0.565
Kidney stones	15 (93.75)	106 (89.08)	
Ureteral stones	1 (6.25)	13 (10.92)	
Stone size (Maximal diameter, cm)	3.02 ± 0.84	1.24 ± 0.45	<0.001
Length of hospital stay (day)	7.94 ± 3.05	6.54 ± 1.82	0.129
Operation time (min)	115.19 ± 46.74	61.40 ± 26.82	<0.001
Intraoperative blood loss (ml)	30.00 ± 16.49	1.83 ± 1.31	<0.001
Preoperative serum creatinine (umol/L)	116.63 ± 105.04	73.41 ± 22.20	<0.001
Preoperative sodium (mmol/L)	140.07 ± 1.72	140.27 ± 1.72	0.432
Preoperative potassium (mmol/L)	4.09 ± 0.28	4.32 ± 3.39	0.586
Preoperative calcium (mmol/L)	2.38 ± 0.30	2.37 ± 0.30	0.392
Preoperative systolic blood pressure (mmHg)	124.88 ± 7.09	127.75 ± 8.24	0.432
Preoperative diastolic blood pressure (mmHg)	77.00 ± 4.08	77.84 ± 6.26	0.174

Univariate logistic regression identified stone size (*p* < 0.001), operation time (*p* < 0.001), and preoperative serum creatinine (*p* = 0.023) as risk factors for postoperative bleeding after R-FURS. Multivariate logistic regression analysis revealed that stone size (*p* < 0.001) and surgical time (*p* = 0.024) were independent risk factors for postoperative bleeding (Table 4).

**Table 4:** Univariate and multivariate logistic regression analysis for bleeding.

Variable	Univariate			Multivariate		
	OR	95% CI	*p*-value	OR	95% CI	*p*-value
Mean age	−0.007	0.954–1.034	0.728			
BMI	−0.143	0.729–1.030	0.105			
Gender	0.969	0.712–9.756	0.147			
Hypertension	0.182	0.391–3.685	0.750			
Diabetes mellitus	0.288	0.354–5.016	0.670			
CHD	−0.205	0.096–6.893	0.851			
Stone location,	0.610	0.224–15.092	0.570			
Length of hospital stay	0.610	0.224–15.092	0.570			
Stone size	3.037	6.074–71.551	<0.001	2.857	4.175–72.650	<0.001
Operation time	0.033	1.019–1.048	<0.001	0.029	1.004–1.054	0.024
Preoperative serum creatinine	0.017	1.002–1.033	0.023	−0.002	0.980–1.016	0.808
Preoperative sodium	−0.067	0.691–1.265	0.664			
Preoperative potassium	−0.040	0.711–1.299	0.796			
Preoperative calcium	0.100	0.217–5.638	0.904			
Preoperative systolic blood pressure	−0.048	0.888–1.024	0.188			
Preoperative diastolic blood pressure	−0.025	0.891–1.069	0.602			

## 4 Discussion

Most urinary tract stones require surgical treatment, with approximately 22% of upper ureteral stones passing naturally (12). R-FURS has become a widely applied, minimally invasive, and effective method for treating upper urinary tract stones (13). Although this method has achieved good results in treating upper urinary tract stones, postoperative complications still pose certain risks.

In the last two decades, advances in medical devices for endourological surgery have made R-FURS widely used, but the occurrence of complications has attracted more attention. Postoperative UTI and bleeding are the most common complications of RIRS. The incidence of significant postoperative bleeding is about 0.1%–2% (14), while mild bleeding, such as hematuria, occurs at a higher rate (10% to 20%) and is mostly self-limiting, not requiring special treatment (15). Omar et al. (15) found that 87.7% (64/70) of patients undergoing holmium laser lithotripsy for ureteral stones achieved stone clearance, with 4% experiencing mild bleeding. The postoperative UTI rate was 6.3%, and the sepsis rate requiring intensive care was 1.3% (14). In this study, the postoperative UTI rate was 8.15% (11/135), and the postoperative bleeding rate was 11.85% (16/135), which is consistent with previous studies.

Among PCNL, ESWL, and R-FURS, PCNL generally has a higher complication rate, followed by R-FURS, while ESWL has the lowest. However, in Kartal et al.'s study (16), no significant difference was observed in complication rates between R-FURS and ESWL in the first 15 days after surgery (*p* = 0.066); however, by the third month, significant differences were noted (*p* = 0.022). This difference was attributed to a higher complication rate in ESWL compared to R-FURS. For experienced surgeons, R-FURS may achieve a lower complication rate compared to ESWL due to its more precise operation, better stone removal, and less damage to surrounding tissues, while the high-energy shockwaves from ESWL can cause more damage to surrounding tissues, making it harder to avoid.

In this study, it was found that high preoperative creatinine levels are risk factors for postoperative urinary tract infection and bleeding. This may be related to the following factors: (a) An increase in creatinine usually indicates impaired renal function. When there is renal insufficiency, the body's metabolic products cannot be effectively excreted, and some substances that affect blood coagulation function, such as guanidines, phenols and other toxic substances, will accumulate. During the operation, it is prone to cause bleeding, and the wound surface is not easy to stop bleeding after the operation, increasing the risk of postoperative bleeding. (b) When there is renal insufficiency, the overall nutritional status of patients is often poor. The metabolic disorder of nutrients such as proteins will affect the normal function of the body's immune system, leading to the inhibition of the generation, differentiation and function of immune cells, a decrease in the level of immunoglobulins, and a weakening of the body's immune defense ability. Therefore, patients are more likely to be invaded by pathogens such as bacteria after the operation, increasing the probability of developing urinary tract infections. The assessment of preoperative renal function is, to a certain extent, beneficial for clinicians to judge the occurrence of postoperative urinary tract infection and bleeding.

There is currently no clear conclusion regarding the risk factors for the occurrence of complications after R-FURS. In this study, it was found that the size of the stone is an independent risk factor for infection after R-FURS, and the size of the stone and the operation time are independent risk factors for bleeding after R-FURS. The multivariate logistic regression analysis conducted by Shimpei Yamashita et al. (17) showed that female gender (*p* = 0.02) and the presence of multiple stones (*p* < 0.01) are independent and significant predictive factors for postoperative febrile urinary tract infection. The presence of multiple stones has been reported in multiple studies as a predictive factor for infectious complications in URS cases (18, 19). In this study, all the included patients had single stones, and the impact of multiple stones on postoperative urinary tract infection was not analyzed. In the study by Francesco Prata (20), it was found that the diameter of the stone, the number of stones, the type of ureteroscope, and the operation time are important predictive factors for postoperative urinary tract infection. The operation time and the type of ureteroscope are independent predictive factors for postoperative urinary tract infection. In the study by Peng et al. (21), it was found that gender, age, diabetes, stone diameter, urethral catheter insertion time, and operation time are independent risk factors for urinary tract infection after R-FURS. This is the same as the finding in this study that the size of the stone is a risk factor for infection after R-FURS. For larger stones and multiple stones, a longer operation time is required to break up or remove the stones during R-FURS. This will lead to increased irritation and trauma to the urinary tract and surrounding tissues, thus increasing the risk of postoperative bleeding and UTI. At the same time, when the stones are larger or there are multiple stones, their surfaces may carry bacteria or pathogens of UTI. During the operation, the rupture of the stones may cause these bacteria to enter the urinary tract, thus triggering an infection (22). Clinical guidelines recommend endoscopic intervention for kidney stones no larger than 20 mm in size and challenging lumbar stones (23, 24). This may also be due to concerns that overly large stones are likely to lead to the occurrence of postoperative complications.

There are relatively few studies on bleeding after R-FURS, and most of them focus on the analysis of the risk factors for bleeding after PCNL (25, 26). The study by Han et al. (27) found that for the treatment of kidney stones by PCNL, the independent risk factors for postoperative bleeding are multiple stones (*p* = 0.008) and stone size (*p* = 0.014). In the study by Tan et al. (28), severe bleeding after PCNL is associated with lower calyx puncture, multiple kidney stones, and single kidney stones. However, the general cause of bleeding in PCNL may be related to puncture. The bleeding after R-FURS is more closely related to the nature of the stones. In the study by Carlo Giulioni et al. (14), 6669 patients underwent R-FURS, and 5.5% of the patients required blood transfusion due to bleeding after the operation. The patients who needed blood transfusion generally had larger stone diameters, especially among those with stones exceeding 20 mm. In this study, it was found that stone size and operation time are independent risk factors for bleeding after R-FURS. There may be the following reasons. Larger stones require longer operation time and more complex operations, which not only increase the mechanical damage to the urinary tract but may also lead to urinary tract obstruction, fragment retention, and local inflammatory reactions, further increasing the risk of postoperative bleeding. Prolonged operation may lead to further damage to the urethral and ureteral mucosa, increasing the possibility of blood vessel rupture.

In fact, there may be many influencing factors for UTI and bleeding after R-FURS, which are not all covered in this article. The placement time of the ureteral stent before the ureteroscopy may be strongly correlated with UTI (29). The use of antibiotics also determines the occurrence of UTI to a certain extent. Factors such as the surgeon's operative experience and the choice of the endoscopic sheath during the operation are all related to bleeding. These may all be aspects that clinicians need to pay attention to.

Although the risk factors analyzed from different data sources are different, this still has clinical significance. It provides a certain reference for optimizing patient management, reducing complications, and improving the postoperative recovery effect. By understanding and addressing these risk factors, the treatment effect can be further improved, the incidence of postoperative complications can be reduced, and thus the safety and quality of life of patients can be enhanced.

This study has certain limitations. First, due to its retrospective design, some key indicators that may affect postoperative urinary tract infection and bleeding are missing, such as ureteral stent dwell time, antibiotic prophylaxis or treatment, and stone composition. May affect the comprehensiveness and accuracy of the results. Second, as a single-center study, the sample source is relatively homogenous, and regional bias may exist, limiting the external applicability of the results. In this study, the numbers of cases with infection and bleeding as the outcomes are relatively small. To a certain extent, there may be some bias in conducting statistical analysis. Furthermore, the retrospective nature of the data collection may lead to incomplete or inconsistent variable recording, which could affect the reliability of the conclusions.

## 5 Conclusion

Stone size is an independent risk factor for postoperative UTI after RIRS. Stone size and surgical time are independent risk factors for postoperative bleeding.

## Data Availability

The data analyzed in this study is subject to the following licenses/restrictions: the dataset for this study is from our center and is not currently available for external sharing. If any readers wish to access the dataset, they can contact us later. Requests to access these datasets should be directed to 1589259358@qq.com.
